# UK national newspapers’ representation of cancer patients and their care during the early part of the COVID‐19 pandemic: Invoking and undermining solidarity?

**DOI:** 10.1111/1467-9566.13790

**Published:** 2024-05-25

**Authors:** Betsy Barkas, Anne Kerr, Gavin Hawkton, Claire Kish

**Affiliations:** ^1^ School of Social and Political Sciences University of Glasgow Glasgow UK

**Keywords:** cancer, COVID‐19, media, National Health Service (NHS), solidarity

## Abstract

During the COVID‐19 pandemic media narratives of solidarity often cast nations like the UK as if at war, while acclaiming health‐care workers as heroic and beloved. However, this solidarity was often fragile and fleeting, as concerns and criticism about workers, citizens and services also circulated. In this article we explore these dynamics of solidarity in more depth, analysing framings of cancer patient suffering, private and public provision of health care in news media during the early part of the COVID‐19 pandemic. We explore how cancer patients were positioned as victims of failure and abandonment by the state and its health‐care providers, and how the private health‐care system was presented in a saviour role. We conclude by reflecting on the implications of new media’s alignment of appeals to solidarity with highly individualised forms of care and the consequences for state‐based services founded on principles of solidarity.

## INTRODUCTION

During COVID‐19, media narratives of solidarity often cast the nation as if at war, celebrating mutual aid, essential workers and fundraising by heroic figures like the UK’s Captain Tom (Browning & Haigh, 2022; Gui, 2021). Yet these nostalgic celebrations of camaraderie proved to be fragile and fleeting, as a range of morally reprehensible subjects emerged, including asymptomatic spreaders and careless lockdown flouters (Hier, 2021; Skog & Lundström, 2022). Meanwhile, working conditions and resources for essential services deteriorated; indeed, some have argued that the centring of health‐care ‘heroes’ provided ideological cover for neoliberalism’s ongoing structural violence against marginalised groups and the risks shouldered by health‐care workers (Lohmeyer & Taylor, 2021; Wood & Skeggs, 2020). There was an international flourishing of health‐care crowdfunding during this period, whose narratives emphasise ‘individual struggling’ rather than ‘social suffering’ (Kenworthy et al., 2023) and where the already‐better resourced were more able to mobilise accounts of their pain and suffering to advocate for care. The lauding of the National Health Service (NHS) for its heroism and selflessness also took place within a context that had seen long‐standing criticism for poor standards of care and failures to live up to the founding principle of care provided by the state at the point of need (Barry & Greenhalgh, 2019; Eilenberg, 2018; Walker et al., 2021).

In this article we explore these tensions further, using the lens of solidarity to investigate how cancer patients and their care were framed by the media during the early part of the pandemic. Drawing on the work of Prainsack and Buyx (2012) we define solidarity as arising from the ‘willingness… to assist others with whom a person recognises sameness or similarity in… a relevant respect’ (p. 346), exploring its forms and how it was both evoked and troubled. Guided by Prainsack and Buyx’s (2012) categories of interpersonal, group and institutional (contractual) solidarities, we explore the dynamics and durability of solidarity in media accounts. We focus in particular on the ways in which institutional solidarities were evoked and undermined over the course of the pandemic. Here we attend to the findings of Kieslich et al.’s (2023) interview‐based study across eight European countries during the pandemic where an initial wave of enthusiasm saw publics ‘drunk on solidarity’ (Prainsack, 2020) but was replaced by exhaustion and a longing for state‐led institutionalised forms of solidarity. At the same time we consider how institutional solidarities were framed as endangered when resources were overstretched and universal health care free at the point of need came under pressure.

We focus on cancer patients because they were badly affected by delays and changes in the priorities of care over the pandemic, as the state and the private sector reconfigured to divert resources towards the most urgent needs (Riera et al., 2021). It is notable that the UK’s private health‐care capacity was bought up by the NHS, initially to treat COVID‐19 patients, but as time went on it was used to deliver non‐COVID care including to cancer patients (Lay, 2020). However, the delivery of cancer care in both public and private settings during this period was problematic (Friebel et al., 2022; Greco et al., 2020). Cancer diagnosis rates declined, partly driven by patients not presenting to their GPs, resulting in some private facilities subsequently going into liquidation as the flow of patients anticipated did not materialise (Wise, 2022).

Through an analysis of how the media framed these developments and patients’ experiences, we demonstrate that the news stories in our sample amplified stories of individual patient suffering and bravery, but also bolstered a pre‐existing set of press tropes about a struggling, bureaucratic and abandoning NHS that was failing to live up to its solidaristic principles, lauding patients as consumers and presenting the private sector as a saviour. This draws our attention to the role of the media in curating solidarities: advancing and undermining different forms of solidarity as part of a wider set of social practices. Our analysis also demonstrates the contradictory ways that institutionalised solidarity was both invoked and undermined through a focus on patient suffering. We show that media calls for solidarity with vulnerable and deserving patients challenged with barriers to accessing health care but also aligned with highly individualised forms of care as opposed to the state‐based services that constitute institutionalised solidarity.

In what follows, we look at the backdrop of media coverage of the NHS and cancer patients in the UK and the dynamics of solidarity therein. We then present our methodology and dataset of UK newspaper stories, drawing on approaches to media framing and affective repertoires. This is followed by our findings, showing how the UK national press advanced and valorised some visions and forms of solidarity, while undermining others. In exploring these dynamics, we do not seek to negate patient suffering or charitable campaigns bringing it to the fore, but to understand how these can be taken up in media accounts to press agendas which might run counter to their expressed interests in protecting the NHS. Like many of our readers we have people with cancer in our lives, including those let down by NHS care, and we know suffering is real. Rather than shying away from this we want to understand how patient suffering is being framed within wider public discussions, including in ways that might be uncomfortable for patients and their relatives, but are nevertheless important to understanding the dynamics of contemporary health care.

### Background: Media, NHS and cancer patients

Though not received uncritically by audiences, media representations play an important role in framing and legitimating particular agendas for health care (Briant et al., 2011; Philo, 1996). In the UK the NHS founding principles of universality and comprehensiveness have retained widespread popularity (Stewart, 2023) and are a key repository of ‘weepy national sentiment’ (Fitzgerald et al., 2020, p. 1162). But these principles have come under strain as the NHS moves to a marketised model, characterised by competitive funding and the commodified production of health care (Ruane, 1997). Scholars have argued that media representations of the NHS during this time have enabled these ‘privatising developments’ (Ruane, 1997; see also Barry & Greenhalgh, 2019; Lewis et al., 2018), framing the NHS as in need of reform because it is strapped for cash, inefficient and failing to deliver adequate or universal care. For example, Walker et al. (2021) identified a ‘sensibility’ in UK press reports of a ‘slow burn’ of negativity about a ‘failing’ NHS during the introduction of the Health and Social Care Act. Eilenberg (2018) found that stories of patient suffering played a central role in framing the NHS as villainous and the government as heroic in managing the Mid Staffordshire hospital scandal (2009–2010). Stories of patients being denied care through ‘postcode lotteries’ are common (Abelson & Collins, 2009). Here we see the institutional solidarity of the NHS brought into question.

Alongside these changes, a flourishing voluntary sector of charitable health organisations has emerged to raise the profile of, and advocate on behalf of, specific patient groups (Baggott & Jones, 2014). Patients accrue various forms of strategic resources, including raising the profile of ‘their’ condition, advocating for access to treatments, developing networks of funding and influence both through and outside established charities, drawing on traditional and digital media (Kerr et al., 2021; Petersen et al., 2019). Through such activities these ‘access advocates’ enact group solidarity, but have drawn criticism for a tendency to align with industry interests, given an emphasis on challenging barriers to treatments and trials while media amplifies messages of hope about cancer therapies and cure (Davis, 2015). A different form of solidarity is evoked by the more radical history of patient campaigns that destabilised and reworked both existing conditions and established practices, as happened in AIDS activism’s citizen scientists but also within breast cancer activists’ challenging of the environmental causes of breast cancer (McCormick et al., 2003; Petersen et al., 2019). These criticisms draw our attention to the ways that dominant forms of contemporary patient advocacy may advance some forms of solidarity while sidelining others.

We can trace these different solidarities across media accounts, but note there is a predominant framing of patients in individual terms, often focusing on mediagenic, younger ‘desperate’ patients seeking last hopes and miracle treatments in the face of cold or distant bureaucratic public health officials (Abelson & Collins, 2009; Gabe et al., 2012). This draws from a wider repertoire of metaphors and narratives prominent across media and popular culture, including that cancer is a deadly killer, cancer invades the body, and biomedicine and/or patients are engaged in a war with cancer (Clarke & Everest, 2006). Cancer patients are framed as the victims of unpredictable intrusion or attack on the entitlements of childhood or family life (Dixon‐Woods et al., 2003). The image of young, hopeful, defiant and courageous patients battling cancer is common (Burles, 2018; Clarke & Everest, 2006; Seale, 2003), crystallised in the figure of the breast cancer survivor, who has become the ‘archetypal’ cancer patient and synonymous with consumerist ‘pink ribbon culture’ (Bell, 2014; Sulik, 2010). There are important affective dimensions within this repertoire: cancer is conflated with fear, and hope resides in cure (Clarke & Everest, 2006). Typically, audiences are positioned to recognise similarities between their own lives and the patient through an emphasis on a relatable, ‘normal’ and ‘respectable’ life disrupted by an unpredictable outside force. A long‐established finding of the literature is that audiences are positioned as a self‐responsibilised patient‐consumer who is vigilant and prudent about their own health through the consumption of technoscientific messages about cancer signs, symptoms and risks (Burles, 2018). These underlying media scripts of cancer as intrusive and unpredictable, and cancer patients as deserving and relatable, also present opportunities for entrepreneurial patients who can strategically use media for accruing various forms of resources either for individual benefit or on behalf of patient collectivities (Kerr et al., 2021).

Taking these invocations of vigilant self‐actualising patients alongside a backdrop of criticism of the NHS as failing to live up to the ideals of institutional solidarity on which it was founded, we can see that NHS and cancer patient solidarities were far from straightforward or uncontested, even before the pandemic struck. This raises the question of what happened when the pandemic placed even more pressure on cancer patients and services: was institutional solidarity privileged in media accounts, or weakened, and how did this relate to the evolution of the private sector and the kinds of patient campaigns and collectives that were prioritised?

## METHODOLOGY AND DATASET

To investigate these issues we analysed coverage in national newspapers given our interest in how these narratives relate to national health‐care policies and debates, and the long‐running criticism of ‘crisis‐ridden’ public health care in the UK press (e.g. Barry & Greenhalgh, 2019; Lewis et al., 2018; Walker et al., 2021). We did not have capacity to study the wider array of online content produced by patient‐advocates and voluntary organisations, but our sample included the interventions of prominent patient‐influencers curating a presence across multiple platforms, given that legacy media content is also conditioned by social and digital media (Philo et al., 2015).

We drew on framing analysis, which attends to the ways that issues can be conceptualised in different ways through foregrounding some aspects and eliding others, exploring how this aligns with the strategic interests of various actors in advancing authority, power or resources (Hawkins & Holden, 2013). Frames can be characterised by keywords, stereotyped images, sources of information, affective dimensions and thematically reinforcing clusters of facts and judgements dimensions (Entman, 1993, p. 52). We were particularly interested in capturing affective dimensions and metaphors, and symbolic dates and statistics as key topics of interest in the abovementioned literatures on cancer news and in studies of health crisis reporting (Billig, 2021; Craig, 2020). We chose a timeframe of March 2020–January 2021 to capture coverage of the first and second national UK lockdowns and so we could gauge any shifts in framings.

We obtained our sample from the Nexis database using keywords in a search string to capture articles about cancer treatment and COVID‐19. We were interested in analysing news articles with substantial discussion of cancer care, hence included only articles of at least 500 words. Table 1 shows the breakdown of the sample: we included five UK national newspaper titles, selected to ensure a spread across the left–right political spectrum and markets and including Sunday editions: on the right we have *The Daily Mail* and *Mail on Sunday*, *The Times* and *The Sunday Times* and *The Sun*, and on the left we have *The Guardian* and *The Observer*, *The Mirror* and *The Sunday Mirror*. Duplicate and irrelevant (e.g. incidental mentions of cancer) content was removed manually. The total number of articles included in the dataset was 422. The balance of broadsheet, midmarket and tabloid outlets was roughly equivalent: tabloid account for (*n* = 182) 43% of the total, broadsheet account for (*n* = 173) 41%, and midmarket 16%. Articles from right‐of‐centre leaning publications (*n* = 186) account for 44%, and those from left‐of‐centre leaning publications (*n* = 236) account for 56% of the total.

**TABLE 1:** Breakdown of newspaper articles sampled.

Newspaper title	Political orientation	Market	Number of articles	% Share of total
The Guardian and Observer	Left of centre	Broadsheet	83	20
The Times and The Sunday Times	Right of centre	Broadsheet	90	21
Daily Mail and Mail on Sunday	Right of centre	Midmarket	67	16
The Sun	Right of centre	Tabloid	29	7
The Mirror and The Sunday Mirror	Left of centre	Tabloid	153	36
Total			422	100

The data was analysed in an inductive process that involved a gradual refinement of codes during successive readings of the data. We identified some initial topics of prominence based on a first reading: these included patient experience of disruption to cancer care and the role of the NHS, government and the private sector, cancer patients ‘shielding’ at home and the COVID‐19 vaccine. These key topics became the first coding framework that we applied using NVivo. Having applied this first set of codes, we were then able to identify the most important themes and the connections between the themes. Based on our discussion of these connections and overlaps, we identified four main frames, each dealing with a distinct thread of the overarching story of the crisis and each characterised by a particular affect: vulnerability and deservingness, abandonment, institutional failure and hope. These four frames became a second coding framework, which we also applied to the dataset in NVivo: our review of the literature enabled us to then refine the themes in relation to the analysis of solidarity above, and we present the results in the sections below.

## FINDINGS

### Overview

The impact of the pandemic on cancer care and patients was widely reported from March 2020 onwards. Cancellation of surgeries and diagnostic tests and pauses in screening programmes were among the first big news stories, along with experiences of shielding and lockdown: most were stories of crisis. The NHS launched two campaigns ‘The NHS is open for business’ and ‘Help Us to Help You’ in April and October 2020, respectively, both encouraging patients to seek help for symptoms and attend hospital. However, this was countered by numerous reports of patients unable to access treatment (e.g. Cancer patient's family accuses Boris Johnson of ‘hoodwinking’ UK in coronavirus promise, *Mirror*, 6 May 2020). Key moments included a July 2020 British Broadcasting Corporation (BBC) Panorama programme, *Britain’s Cancer Crisis*, presented by cancer patient and *Sun* columnist Deborah James raising the alarm about projected numbers of deaths from cancer, and a leak of NHS data in September 2020 showing thousands of cancer patients were waiting for months for tests or treatment, though this figure was disputed by the NHS (Waiting list for cancer specialists up by a fifth, *The Times*, 30 September 2020).

Patient testimonies were especially prevalent within the *Daily Mail*, *Sun* and *Mirror*, while the *Guardian* and the *Times* focused more on pressures on the NHS as an institution. The theme of abandonment featured most heavily in the *Daily Mail*. Echoing findings summarised above, press reports were unrepresentative of wider demographics of cancer patients and emphasised younger patients and particular types of cancer. Reflecting her longstanding advocacy and social media presence Deborah James was a highly visible figure during this period; after the time period sampled she was made a Dame having raised over £6 million for her charity Bowel Babe Fund before her death in 2022 (Rogers, 2022).

### Vulnerability and deservingness: Calls for solidarity with an abandoned group

Initial coverage reproduced elements of the UK government’s official messaging identifying vulnerable groups and exhorting the public to ‘Protect the NHS’. Cancer patients were established as vulnerable and deserving of care, initially in contrast to a second group of immoral lockdown ‘flouters’. Stories featured a ‘desperate’ teenage cancer patient who said ‘Stay at home or you could kill me’, and the mother of a child cancer patient who urged the public to ‘[take] this seriously… We’re all in this together and we need to think about the most vulnerable…’ (Cancer patient, 18, issues desperate plea to lockdown flouters saying ‘you'll kill me’, *Mirror*, 25 March 2020; Mum of dying boy, 3, forced to spend last days in isolation makes heartbreaking plea, *Mirror*, 8 April 2020). Deborah James’ *Sun* columns invoked an embattled ‘cancer community’, as in a March 2020 column where she addressed directly ‘selfish’ people who were endangering both cancer patients and the health system itself by ‘going to the pub’ (Deborah James: I’m being tested for coronavirus—which could kill me—while selfish people go to the pub’, *The Sun*, 20 March 2020). These articles carried an implicit framing of readers as morally righteous health‐care users being asked to play their part through complying with lockdown restrictions, while the newspapers positioned themselves on the patients’ side, defending the underdog. Those perceived to be acting without solidarity were shamed and castigated, positioned as distant ‘others’ whose possible motivations for their ‘selfishness’ were left unexplored.

However, this early emphasis on consensus about social distancing and endorsement of the government’s approach eroded over time and the narrative about ‘lockdown flouters’ also disappeared, replaced by a more complex picture. Cancer patients continued to be framed in terms of their vulnerability and deservingness but emphasis shifted to how this group had been deprioritised in favour of COVID‐19 patients. A patient with bowel cancer whose planned diagnostic procedure was postponed indefinitely told the *Guardian*:

I am totally gutted to think I’ve been chucked on the pile in favour of coronavirus victims. For all I know they are giving me a death sentence [by] not looking at my case.

(Coronavirus crisis could lead to 18,000 more cancer deaths, experts warn, *The Guardian*, 28 April 2020).

In September Deborah James spoke out in her *Sun* column:

Yes infections are starting to rise again, but hospital admissions and deaths don’t appear to be spiralling out of control. We absolutely have to stay vigilant and follow the rules… But we need to wake up to the fact that other sh*t is going down too! Last week, on a day when England’s daily COVID death toll was three, I said goodbye to four young friends. They all died of cancer, on the same day… Where is their headline? Where is their daily press conference? Where is there emergency funding to pump cash into their treatment? Where is their global race for a vaccine?

(On a day when 3 people in England died of COVID I lost four friends to cancer—it’s time for some perspective, *The Sun*, 21 Sept 2020).

During the pandemic, announcements of precise numbers of deaths provided a national spectacle through daily publication and featuring in press conferences; here, James challenges what is elsewhere being presented as a successful ‘good number’ (Billig, 2021) of three COVID‐19 deaths with a quantification of her own personal death toll of four friends. Here, the numbers became a rhetorical resource (Potter, 1996) to construct a weighing of the needs of vulnerable cancer patients against those of COVID‐19 patients, also echoing interventions by cancer charities urging action on ‘the forgotten C’, explored in more detail below.

Here we see expressions of anger and frustration as precious time and lives are lost due to treatment delays, alongside the pitting of one group of patients against another—an appeal to solidarity between and with cancer patients but not with other kinds of patients with COVID‐19, whose perceived prioritisation is criticised as unfair. Moralising discourses that construct a boundary between deserving and undeserving health‐care subjects are a feature of pandemic media reporting in general: in Skog and Lundström’s (2022) study, Swedish news media featured reckless elite folk devils, either spreading the virus through unnecessary skiing holidays and parties, or claimants of ‘unnecessary’ health‐care services including cosmetic surgery and caesarean sections. Here, a deserving cancer patient group with inalienable right to treatment is evoked and the audience is called upon to recognise and participate in solidaristic acts at the group level: firstly to protect cancer patients from infection by foregoing the pub, but subsequently to participate in outrage at their unfair treatment. The urgency of solidarity through social distancing compliance was displaced by the call for solidarity through challenging the ‘focus’ on COVID‐19; the out‐group of rule‐breakers replaced by the out‐group of COVID‐19 patients, unfairly monopolising resources. This shift was accompanied by calls for a more institution‐led and institutionalised form of solidarity, including medical research, media coverage and political will and resources to meet the collective needs of cancer patients, both current and potential.

A sense of cancer patients’ abandonment pervaded much of the coverage and was a strong theme particularly in right‐leaning press during the inter‐lockdown period in the summer months when COVID‐19 infection rates were relatively low and before a second wave began to raise fears. In July 2020 Deborah James hosted a programme for BBC’s *Panorama* investigating the impact on cancer patients, reported in a *Times* article in which one of the featured patients expressed anger ‘that the health service was not able to manage a health crisis without creating another one’, and Deborah James agreed ‘one hundred per cent’ (Between COVID and cancer it’s a scary place to be, *The Times*, 6 July 2020). These stories sometimes used the military term ‘collateral damage’, casting cancer patients as abandoned civilians. The anger against the health service was most clearly crystallised in the concept of the ‘COVID‐obsessed NHS’, and the ‘National COVID Service’, two phrases that became prevalent. In September, the *Daily Mail* published a story based on a Freedom of Information request to English hospital trusts that obtained what it called ‘a dossier of complaints’ made by over 200 cancer patients; this story was entitled ‘Cancer Patients’ Corona Betrayal’ (21 September 2020). There were also examples of patients who expressed anger at the government for ‘[giving] a death sentence by stopping treatments’, and ‘concentrating too much on COVID, and not on other illnesses’ (Man, 22, with skin cancer ‘given death sentence’ as treatment cancelled over coronavirus, *Mirror*, 27 April 2020; Op delay timebomb, *Mirror*, 26 August 2020). Charity interventions echoed this division through campaigns such as ‘the forgotten C’ (e.g. Cancer patients must not be forgotten in pandemic, say charity, *The Guardian*, 24 April 2020), reinventing a prominent trope in cancer campaigning and representing a strategic bid for resources within an environment already competitive but made even more so by pandemic conditions (Baggott & Jones, 2014; Butler & Morris, 2020).

Within this frame there was some diffusion of blame in that some patients emphasised support for the NHS and directed anger at the government, particularly within the left‐leaning press. This perspective of government responsibility tended to be contained within quotes from individual patients, whereas the outrage towards the NHS was much more fully developed through a repertoire of betrayal by a health service characterised as ‘COVID‐obsessed’ and a failing bureaucracy, as discussed below. In a marked contrast to the displays of love and support for the NHS elsewhere, there were few positive depictions of the health service within our sample: these few tended to be framed in terms of ‘innovations’, for example, the use of ‘COVID‐free’ hubs, or the new working arrangements with the private sector, discussed below, rather than stories of continuing care; in fact, the more complex picture of ongoing day‐to‐day provision of cancer care to patients was largely absent from the coverage. These narratives further rework solidaristic notions about the NHS and patients, framing cancer patients’ collective pain and suffering as having gone unnoticed or being written off as collateral damage for the wider population’s benefit. As the pandemic advanced, the calls for solidarity with this vulnerable group were intensified in ways that were more overtly critical of the health service, which we explore further in the following sections.

### Dread and collapse: Failures of institutional solidarity

While official NHS statements to the press exhorted the public to seek help for any concerning symptoms and emphasised that the health service was ‘open for business’, the overriding framing of the NHS was as a service failing to deliver. In places we found that this framing was more fully developed through a narrative of institutional failure, drawing on a rhetoric of decay and collapse. Sometimes coverage of the NHS included apocalyptic framings of ‘crisis’ and ‘nightmare’ (3BN OP BACKLOG, *Daily Mirror*, 26 April 2020; CANCER NIGHTMARE; CORONAVIRUS CRISIS, *Daily Mirror*, 29 October 2020). Metaphors of structural collapse and damage to the health service featured prominently: ‘stretched hospitals’ were starting ‘to buckle’, while the NHS was ‘bursting‐at‐the‐seams’ (50,000 CANCER CASES MISSED, *Daily Mail*, 29 October 2020; Coronavirus changes cancer treatment for mum told it had been caught ‘early enough’, *Daily Mirror*, 9 August 2020). There were also metaphors of natural or elemental force or scale, as when the NHS was described as having an ‘enormous mountain to climb’ to clear the screening backlog (A MILLION WOMEN MISS VITAL BREAST SCREENING, *Daily Mail*, 30 September 2020). An April 2020 *Times* editorial described the collective of patients as a ‘wave’:

Once the NHS staggers out of this pandemic, its staff will be shattered and its resources depleted. The last thing it will need is a new wave of patients, by now desperate for the treatment they so deserve.

(Cancer treatment will be the next NHS crisis, 15 April 2020)

In an April *Daily Mail* editorial, cofounder of private oncology firm Rutherford Health (which went into liquidation in 2022 following a reduction in patient numbers presenting to its clinics) Karol Sikora wrote that:

[T]he cancer diagnostic system has all but seized up. Another 400 cancers a week are, it is estimated, being missed because breast, cervical and bowel cancer screening has been suspended. For any of these patients, delay can be a death sentence. In addition, the NHS has cancelled most routine surgeries for three months. That’s tens of thousands of people who must endure further pain and distress as they wait for the operation or procedure that might dramatically improve their quality of life. All of these individuals are collateral damage in the war against coronavirus. […] Even before coronavirus hit, many of Britain’s oncology departments were at breaking point. We don’t like to admit it but access to health is rationed in this country…

(IF THIS GOES ON FOR SIX MONTHS 50,000 MORE PEOPLE COULD DIE OF CANCER, 22 April 2020).

The repetition of tropes of powerless patients, ‘rationing’ and ‘cost‐cutting’ within an ineffectual bureaucracy also aligned with arguments for the alternative organisation of health‐care services. There were several other similar interventions made by Sikora in our sample, the tone and content of which resonated with his own previous forays into the debate over NHS care, including appearing in ads in the United States attacking Barack Obama’s plans to reform US health care, in which he warned that NHS patients ‘lose control over their own destiny in the health system’ (McGreal, 2009). Among the most forthright expositions of these themes within our sample was a *Times* editorial ‘Underlying Conditions: The coronavirus crisis has highlighted fundamental problems in Britain’s system of healthcare. Now is the time to confront them’ (June 2020). It listed problems with the NHS including that it was overflowing, overcrowded, short of staff, and was hamstrung by bureaucracy and inefficiencies including a postcode lottery for cancer referrals.

The narrative of collapse and metaphors evoking risk and danger show a marked similarity to the nationalistic tropes used within right‐wing press in constructions of migrants ‘flooding’ into the country which form part of what van Dijk has called a ‘numbers game’ (Van Dijk, 2000). The extracts above show a similar set of strategies, including the use of round or unspecified numbers (Billig, 2021) such as ‘400 cancers a week’, ‘tens of thousands of people’, as well as the characterisation of patients as a ‘wave’. This evokes a sense of threat at a national level caused by unmanageable and unmet need: the readership is positioned to be not only in sympathy with cancer patients but to have their own feelings of dread as they also depend on an overwhelmed health service. These tropes were aligned with patient stories featuring emotive expressions of exhaustion, fear and powerlessness. For example, quotes by patients included expressions such as ‘life on hold’, and used potent imagery: one described their cancer as a ‘ticking time bomb’, another said ‘“Cancer patients like me were just left, like sitting ducks. The cancer has been eating away at my body. I feel like I’m being murdered, plain and simple”’ (“I’m very frightened”: lives on hold as NHS surgery postponed due to COVID‐19, *Guardian*, 19 March 2020; [A patient], 72, HAD HER STAGE 3 CANCER OP CANCELLED AT THE LAST MINUTE TO MAKE WAY FOR COVID CASES; KILLED, *Daily Mail*, 17 October 2020). Unlike in other studies of cancer news (Clarke & Everest, 2006), here the framing of dread centres not on cancer itself but on unmet health need and a lack of protection: it evokes a sense of dread among cancer patients but also for the reader, positioned to be a patient‐consumer anxious about a collapse of health‐care provision.

Despite numerous calls for investment spearheaded by cancer charities, here the NHS was often framed in terms of flaws, not potential; the scale and complexity of the NHS were foregrounded, while the role and responsibility of the state for organising and funding health care were sidelined. There are resonances here with a wider politics of nostalgia that unfavourably compare a decaying present to a better past, and in which post‐war welfare states figure strongly (Elgenius & Rydgren, 2022). Within wider COVID‐19 discourses the NHS appeared as a treasured national institution symbolically tied to a glory‐saturated post‐war Britain, but here appeared within a construction of a decaying national present characterised by feelings of loss, dread, anxiety and abandonment. This expressed both a longing for institutional solidarity but also anger at the failures of those institutions to deliver on their promises—a theme of betrayal where the focus was not on salvaging institutional solidarity but framing it as a failed project.

### Hope: Seeking solutions

The final theme in our analysis featured solutions to the crisis and built on common framings of cancer patients as heroic and self‐actualising. There were many examples of patients exerting control, including through ‘everyday’ acts such as adapting to lockdown conditions, making the most of limited time and enjoying spending time with family (e.g. Mum whose ‘trapped wind’ was cancer has to break lockdown to tell children she’s dying, *Mirror*, 4 July 2020). However, most patients exercising autonomy and choice were either pursuing private care for their own delayed or cancelled tests, treatments or drug trials, or challenging the NHS through litigation. Within these stories, the determination and stoicism of patients in pursuing ‘only chances’ or ‘last hopes’ was often emphasised, and fundraising efforts drawing from the solidarity of communities were celebrated. For example, in August a story featured the ‘first known cancer patient to become terminal’ after cancellation of treatment, whose ‘family [were] not giving up hope’, who raised an ‘amazing’ amount on a fundraising page for 7 months of Avastin, a targeted chemotherapy drug used for palliative treatment (Man is first known cancer patient to become terminal after coronavirus cancels treatment, *Mirror*, 25 August 2020). Another patient was a ‘brave mum’ who had ‘trawled the internet to find an expert’ (Mum told three times she had acid reflux when it was actually terminal cancer, *Daily Mirror*, 31 August 2020). A *Daily Mail* article in October titled ‘WE ARE CANCER PATIENTS STILL BEING LEFT IN LIMBO’ (6 October 2020) featured more ‘resourceful patient’ stories: about one who had paid privately to have his prostate removed, the article commented: ‘Had he not taken matters into his own hands, he might have faced a different outcome’ (*Daily Mail*, 6 October 2020). A further topic was patients suing the NHS, who were often also crowdfunding for treatment. In one story a patient who had instructed a lawyer to investigate the possibility of suing was described as ‘fighting for life’ (Man ‘fighting for life’ after COVID‐19 crisis delays NHS cancer scan, *The Guardian*, 9 June 2020).

This framing reflected a sense of dread, abandonment and anger outlined within the other frames, but here instead of powerlessness victimhood the active efforts of patients were foregrounded. The two extracts below give examples of stories about patients who had embarked on personal fundraisers.

[The patient] said she was in a “living nightmare”. To find the £160,000 she needed, the former hairdresser… cashed in her £40,000 pension. Her sons set up a Gofundme page. By paying for the treatment herself, she hopes to win more time with her family. [She] said: “I’ve worked hard all my life, I’ve saved in pensions to provide for myself when I’m older and basically I am absolutely appalled … the system has failed me”.

(‘Cancer sufferers go door to door to pay for private treatment’, *Sunday Times*, 27 September 2020)

In the meantime, [the patient] is pinning her hopes on raising the money to buy her chance to live’. So far, £89,000 has been raised enough to begin treatment. Her first session will start imminently. She says: “The NHS has let me down. It will only be thanks to the generosity of so many others that I may have a fighting chance”.

(‘COVID’S CRUEL BLOW TO CANCER LIFELINE’, *Daily Mail*, 25 August 2020)

A distinguishing characteristic of the NHS as archetypal solidaristic institution funded through progressive taxation is that one’s contributions reflect their ability to pay. It is a system in which there is a solidarity of lifestyle and income, meaning that treatment is received regardless of ability to pay or ‘unhealthy’ lifestyle (Prainsack, 2018): it is not earned but entitlements derive instead from citizenship. In the extracts above we see the recasting of this principle as a logic of individualised personal insurance, where ‘paying in’ and ‘working hard all one’s life’ earned the entitlement of health care; here the system is portrayed again in terms of a broken promise. Patients were then pushed to seek refuge in the market—though not willingly, as hopefulness was mixed with anger or a sense of betrayal. Patients at times expressed regret about being a private patient, as one former NHS worker conducting a crowdfunder put it: ‘I don’t want to be a private patient, but what choice do I have’ (NHS worker, 49, who has never smoked diagnosed with stage four lung cancer, *Mirror*, 7 September 2020).

However, elsewhere there were positive and hopeful depictions of the growing role of private health care within the provision of care, particularly within the *Times*. For example, a May 2020 profile of a private hospital executive was framed in sentimental terms:

Justin Ash dabs his eyes. The boss of Britain's biggest private hospital group is recalling an emotional goodbye. A patient in her eighties was being treated in a Spire hospital while her husband of 62 years was receiving care for COVID‐19 in a nearby NHS ward.… “When you think about what this really means—people working together during a crisis—I don't think we can go back to the ‘two camps’ approach… For me, that will be what COVID‐19 was all about. NHS to private, nurse to nurse. Everyone was crying”.

(We're all in this together; Links between private and public healthcare forged in the pandemic will live on, says Spire chief Justin Ash, *Times*, May 2020)

The presentation of the NHS as failing to deliver on the promise of institutional solidarity and the private sector as a saviour was threaded through many of the stories celebrating patient autonomy and choice. The figure of the ‘heroic’ patient who remains steadfast in the face of adversity resonates with findings from the literature on cancer news reporting (Burles, 2018), and also reflects the rise of the entrepreneurial patient, treated in media as a brave figure battling the odds to get treatment they were entitled to but denied. Crowdfunders and legal challenges for medical treatment were presented ambiguously, as they emerged out of desperation, but the reader was being invited to identify and root for their success, even while feeling outraged at the necessity. We can commend people for finding alternative routes to meeting health need at times of uncertainty, while also recognising that this challenges solidaristic approaches to health care (Stewart et al., 2022). Here, we found the trope of the ‘last chance’, ‘fighting chance’ and ‘lifeline’ provided privately aligned with an overall depiction of institutional solidarity in the shape of the NHS as a failed ideal. This then created a discursive space for the positive, even sentimental, stories of collaboration between private and public health care found in the right‐leaning press. These themes of hope, lifelines and seeking refuge in the private sector again drew attention to the still existing legal framework underpinning NHS care, but framed this in terms of a longed‐for but failed ideal and broken promise, while the pragmatic and realistic solution was market‐based and individual.

## DISCUSSION AND CONCLUSIONS

Stories about patients seeking care outside the NHS are not a novel feature of the COVID‐19 period, and the figure of the abandoned, ‘desperate’ cancer patient is visible within previous studies of media coverage of access to cancer drugs or cancer screening tests (e.g. Fredheim, 2021; Hind et al., 2011). The NHS has also long been criticised for quality of care at the same time as it is valourised as a great British institution. There were, however, some novel and striking features of the ways that cancer patients and their care were framed within press coverage during the early part of the pandemic. Despite the wider context in which the NHS and its workers were being celebrated, we found that the dominant characterisation in our findings was of patients failed by an institution in crisis. Other studies analysing news coverage around access to specific cancer drugs or screening programmes have identified criticism of public health officials as distant or cold‐hearted bureaucrats (Mackenzie et al., 2007). In our study we observed themes of dread, collapse and abandonment that extended these tropes to present patients at the mercy of a malfunctioning system. Particularly striking were the ways that the structural collapse of the service was linked to an overwhelming surge of patients, sometimes compared to an elemental or natural force, a novel finding that provides a counterpoint to the individualised way that cancer patient experiences usually are conceptualised within news media.

A further original finding is the ways that COVID‐19 interventions were framed as the denial and abandonment of cancer patients by the NHS, rather than a public health intervention aimed at reducing the impact of the pandemic on everyone. This framing elided structural factors, such as historical underfunding or staffing shortages, lack of government preparedness and delayed interventions to mitigate the COVID‐19 crisis, as well as the ongoing provision of care to patients. Instead, stories repeated a prevailing discourse of crisis and even decay. Taken together with other findings about the representation of NHS as mired in crisis and scandal (Eilenberg, 2018; Walker et al., 2021), this points towards an overarching news script (Entman, 1993) in which individual patients and their inalienable rights to treatment are valourised, while simultaneously state health care is presented as a failed ideal. Studies of pandemic media reporting have largely focused on the ways that state power is legitimated including via constructions of a morally righteous and self‐responsible ‘pandemic subject’ (Hier, 2021; Lövenmark et al., 2023; Skog & Lundström, 2022). Looking in depth at what we might call a subsidiary crisis invoked in media coverage of cancer care, our analysis adds a nuance to these findings by showing that within our sample it was a market model of health care that was being celebrated and legitimated, while the authority and status of the state health‐care system was being undermined.

Though we focused on representations of patients and care, our findings also contribute towards understanding how the news media constructed subject positions for their readerships during COVID‐19. Lövenmark et al. (2023) have found dominant reader subject positions of ‘good readers’ who are constantly learning and vigilant subjects, passively absorbing expert knowledge and official regulations, and in this they encourage self‐government and self‐discipline. In our sample of cancer news there were elements of reader self‐responsibilisation in the calls to protect cancer patients through personal adherence to lockdown measures, but this was fleeting: later coverage encouraged readers to support patients by donating to crowdfunders and consuming further content produced by cancer patient‐advocates. There was a new kind of vigilance being introduced in that readers were positioned as morally righteous, sympathetic and in solidarity with vulnerable cancer patients, but also prudent in awareness of a collapsing and failing public health‐care system, and being educated about private treatment options that might represent the ‘last hope’. In telling the story of this crisis, media therefore promoted individualised and market‐based solutions, even while coverage was saturated in a repertoire of ideas about collectivised agendas and needs, and acts of interpersonal and group solidarity.

The rising prominence of patient experiences has brought considerable positive change to health‐care provision, bringing in new kinds of experts and fostering togetherness and support (Frank, 1995). We can see that the more traditional journalistic use of ordinary patients as exemplars is merging with an increasing prominence of patients’ user generated content such as crowdfunders and social media activity, linked to emotionally driven ‘authentic’ perspectives (Wahl‐Jorgensen, 2020). However, we also need to attend to how these selective framings of patient experience in public discourse cohere with other agendas that seek more radical kinds of change, including marketisation and privatisation of health care. Within our sample, two main kinds of cancer patients emerged: abandoned victim and courageous fighter. This recalls tropes in cancer reporting elsewhere (Seale, 2003), but in this case tied to criticism of NHS care. While some patients were quoted criticising the government and defending the NHS, the overall news storyline emphasised institutional failure and a failed promise.

Our findings extend recent studies of the affective and cultural import of the NHS, which has come to represent abstract ideals rather than material realities (Fitzgerald et al., 2020; Stewart, 2023). We have noted that calls for solidarity with deserving groups and the longing for welfare state solidaristic institutions can become strategic resources within a nationalist politics of nostalgia (Elgenius & Rydgren, 2022). Alongside the prominence of war and military metaphors and nationalistic constructions of duty during COVID‐19 the NHS has been undergoing a longer‐term rebranding as a nationalistic achievement (Fitzgerald et al., 2020). During the UK’s 2016 Brexit referendum, ‘Vote Leave’, the official campaign to leave the EU, created an iconic red ‘battle bus’ featuring a slogan on its side: ‘We send the EU £350 million a week. Let’s fund our NHS instead’ (Fitzgerald et al., 2020). In this role as a rhetorical centrepiece for the ‘Leave’ offensive, the affective and symbolic resources represented by the health service were leveraged within a wider politics of anti‐migrant agitation; it is also notable that a likely outcome of Brexit itself is increased profiteering through health‐care privatisation, marketisation and deregulation (Fitzgerald et al., 2020). Here we do not seek to rebut criticism or instantiate our own nostalgic imagery of a health‐care system under attack, but rather to show that in the current political moment we see romanticised and affect‐laden images of solidaristic institutions pressed into service within wider agendas including those that undermine them.

It is important to note that we did not investigate the effects of media frames on public understanding of, or uptake of, health care as this was not the focus of our analysis, and furthermore our suggestion that patients might feel uncomfortable about the use of their stories within framings that arguably undermine the NHS is speculative. However, our study does suggest the need to consider carefully how patient and public perceptions of health care respond to and are shaped by these media frames of patients and care, and how this affects support for, and uptake of, the services provided by institutions and markets. Further work is also needed to investigate the evolving framings of patients, public and private health care arrangements in policy and other public discourses beyond newspapers. We note that since our sampled time period, there has been a continuation of these narratives as press have linked ‘calamity’ and ‘disaster’ in cancer care with the UK’s failures in comparison with other nations (e.g. Pickles, 2024). More work is needed to understand how the analysis presented here may resonate with the changing set of funding and governance arrangements associated with the reorganisation of health‐care provision along market principles.

## AUTHOR CONTRIBUTIONS

**Betsy Barkas**: Conceptualization (equal); formal analysis (equal); investigation (equal); visualization (equal); writing—original draft (lead); writing—review and editing (lead). **Anne Kerr**: conceptualization (equal); formal analysis (equal); funding acquisition (lead); supervision (lead); visualization (equal); writing—review and editing (supporting). **Gavin Hawkton**: Formal analysis (equal); investigation (supporting). **Claire Kish**: Formal analysis (equal); investigation (supporting).

## CONFLICT OF INTEREST STATEMENT

The authors declare no conflicts of interest.

## ETHICS STATEMENT

Institutional ethical approval was granted AREA 15‐108.

## Data Availability

The data that support the findings of this study are available in the public domain from Nexis media archive.
